# Assessment of complications and success rates of Percutaneous nephrolithotomy: single tract *vs*. multi tract approaches

**DOI:** 10.7717/peerj.18450

**Published:** 2025-01-16

**Authors:** Ömür Memik, Onur Karslı

**Affiliations:** Department of Urology, University of Health Sciences Kocaeli Derince Training and Research Hospital, Kocaeli, Turkey

**Keywords:** Percutaneous nephrolithotomy, Complex kidney stones, Multiple tract

## Abstract

**Purpose:**

The objective of this study was to assess the success and complication rates of single-tract access *vs*. multi-tract percutaneous nephrolithotomy (PNL).

**Material and Methods:**

The medical records of consecutive patients who underwent PNL for staghorn, partial staghorn, and complex kidney stones between 2014 and 2022 were retrospectively reviewed. The demographic data, stone volumes, fluoroscopy and operation durations, changes in hemoglobin levels, numbers of blood transfusions, stone-free rates, perioperative complications, duration of nephrostomy removal, and length of hospital stay parameters were noted. The complications and complexity of PNL were graded using the modified Clavien and Guy’s stone scores, respectively. Single and multi-tract subgroups were determined based on PNL access strategy and the subgroups were analyzed.

**Results:**

The study involved 208 patients, with 158 in the single-tract group and 50 in the multi-tract group. The groups were comparable in age, ASA scores, and comorbidities (*p* > 0.05). The characteristics of the stones, such as their location, size, and density, did not show any significant differences between the groups (*p* > 0.05), except for Guy’s stone score, which was higher in the multi-tract group (*p* = 0.028). The multi-tract group also had significantly longer fluoroscopy and operation times (*p* < 0.001). There was no statistically significant difference in stone-free rates between the two groups (76.0% *vs*. 78.0%, *p* = 0.766). Although the drop in hemoglobin levels was more significant in the multi-tract group (*p* = 0.027), transfusion rates did not differ significantly between the two groups (*p* = 0.334). Complication rates were higher in the multi-tract group, but this difference was not statistically significant (*p* = 0.896).

**Conclusion:**

This study demonstrated that multi-tract PNL can achieve high stone-free rates with a modest increase in the occurrence of acceptable complications when performed by an experienced surgeon.

## Introduction

The treatment of staghorn or complex calyceal stones remains one of the most challenging problems in the field of urology (Ganpule & Desai, 2008; Jiao et al., 2020). These stones are usually large and branched and are frequently infected. Staghorn or complex calyceal stones occupy a significant portion of the renal collecting system, including the renal pelvis and multiple calyces. If not treated, they can cause severe renal impairment or sepsis (Ganpule et al., 2020). In patients with complicated or staghorn calyceal stones, the objective of treatment is to ensure maximum clearance of the stones and to preserve maximum kidney function with minimum complications (Jiao et al., 2020). Shock wave lithotripsy (SWL), retrograde intrarenal surgery (RIRS), and percutaneous nephrolithotomy (PNL) are all treatment options for these types of stones, and advances in these treatments have significantly reduced the need for open or laparoscopic stone surgery (Alivizatos & Skolarikos, 2006). PNL is the preferred treatment method because it has higher stone-free rates and lower morbidity than open surgery, especially for complex staghorn calculi (Preminger et al., 2005). Current EAU guidelines list PNL as the standard method of treatment for large kidney stones (Skolarikos et al., 2022).

Achieving stone-free status after PNL becomes more challenging as stone size increases. Depending on the stone burden and the patient’s anatomy, multiple tracts may be required to achieve stone-free status in a single PNL session (Hegarty & Desai, 2006; Huang et al., 2021). Although this approach is widely accepted, establishing multiple percutaneous routes can increase the risk of postoperative complications such as pleural damage, infection, and the need for blood transfusion (Türk et al., 2016; Wang et al., 2021). Therefore, there are ongoing concerns about the safety of multi-tract PNL leading to many urologists having reservations about placing multiple percutaneous tracts during PNL (Jiao et al., 2020; Kukreja et al., 2004).

This study retrospectively compared the perioperative outcomes of patients and assessed the safety and efficacy of PNL with multiple percutaneous tracts.

## Materials and Methods

The medical records of consecutive patients aged 18 or over who underwent PNL for staghorn, partial staghorn, and complex kidney stones at a single center between 2014 and 2022 were retrospectively reviewed for this study. Ethical approval for this study was obtained from the University of Health Sciences Kocaeli Derince Training and Research Hospital’s local ethics committee (2022-111). Patients were excluded from the study if they had impaired kidney function, a history of bleeding disorders, skeletal deformities, a solitary kidney, or anatomical kidney abnormalities such as a duplicated collecting system, horseshoe kidneys, or a ureteropelvic junction obstruction. A staghorn kidney stone was defined as the presence of stones in the majority of the renal pelvis and collecting system. A partial staghorn kidney stone was defined as the presence of stones in the renal pelvis and two or more calyces. A complex calyceal stone was defined as the presence of stones in multiple calyces (Jiao et al., 2020).

Before the surgery, all patients underwent a thorough physical examination and medical history, and routine tests were performed, including blood biochemistry and urine analysis and culture. Both non-contrast and contrast-enhanced abdominal tomography were also carried out prior to PNL. Demographic variables were recorded such as age, gender, body mass index (BMI), and comorbidities including diabetes mellitus (DM) and hypertension (HT). Stone characteristics were noted, including size, location, density in Hounsfield units (HU), and the presence of hydronephrosis. Intraoperative and postoperative parameters, including fluoroscopy and operation durations, changes in hemoglobin levels, numbers of blood transfusions, stone-free rates, perioperative complications, duration of nephrostomy removal, and length of hospital stay were also recorded for each patient. The stone burden of the patients was calculated using the Ackerman formula (volume = 0.6 × π × r^2^), where ‘r’ represents half of the largest diameter of the stone. Additionally, all patients were categorized based on preoperative contrast-enhanced abdominal tomography findings, using Guy’s stone scores (Thomas et al., 2011). Each patient was reassessed by non-contrast computed tomography, which is customarily conducted 1 month after surgery. Operation success was defined as the patient being stone-free or only having remaining stone particles less than 4 mm. The Clavien-Dindo classification system, which has five grades, was used to classify complications (Tefekli et al., 2008).

Each procedure was performed by the same surgeon with extensive experience in the field of endourology. In cases where the surgeon determined that the other calyces could not be reached through a single entrance, a second access point was used during the operation. Patients were divided into single and multi-tract subgroups and the subgroups were analyzed.

### Surgical technique

For the PLN procedure, each patient was given general anesthesia, and an open-ended 5 F ureteral catheter (Marflow^™^, Marflow AG, Switzerland) was inserted with cystoscopy guidance while the patient was in the lithotomy position. After catheter placement, the patient was moved to a prone position, and radio-opaque material and C-arm fluoroscopy were used to visualize the patient’s pelvicalyceal system anatomy. A 19.5-gauge percutaneous needle (Boston Scientific Corporation, MA, USA) was introduced into the appropriate calyx system. Fluoroscopy was used to place a guidewire (Zebra^™^, Boston Scientific Corporation, MA, USA) in the collecting system. The tract was dilated up to 30 F with semirigid amplatz dilators (Boston Scientific Corporation, MA, USA) and an Amplatz sheath was inserted into the collecting system. Stone fragmentation was performed using a pneumatic lithotripter (Calculith^™^ Lithotripter, PCK, Turkey) through a 28 F rigid nephroscope (Karl Storz^™^ Endoscopy-America Inc., El Segundo, CA, USA). Forceps were used to retrieve the stone particles, and the procedures were completed by inserting a 14 F re-entry nephrostomy catheter. For patients undergoing multi-tract PNL, the second entry was introduced using the same methods as the first entry, and a second 14 F nephrostomy tube was placed in the second entry.

### Statistical analysis

Data were analyzed using SPSS Statistics for Windows, version 22.0 (IBM Corp, Armonk, NY, USA). Descriptive statistics were employed to summarize the data, with quantitative variables analyzed according to their distribution. The normality of continuous variables was assessed using the Kolmogorov-Smirnov test. Normally distributed numeric data are presented as mean ± standard deviation (SD), while non-normally distributed data are presented as median and interquartile ranges (IQR). The independent samples t-test was used to compare means between two groups for normally distributed data, whereas the Mann-Whitney U test was used to compare non-normally distributed data. Categorical variables were compared using the chi-square test to evaluate associations between groups and are expressed as frequencies and percentages. A *p*-value of less than 0.05 was considered statistically significant.

## Results

A total of 208 patients who met the inclusion criteria were included in the study, with 158 in the single-tract group and 50 in the multi-tract group. The mean age of the patients was similar in both the single-tract and multi-tract groups (48.6 ± 14.07 years *vs*. 49.2 ± 13.50 years, *p* = 0.798). There was no statistically significant difference between the two groups in terms of ASA scores (median [IQR]: 1 [1–2] *vs*. 1 [1–2], *p* = 0.435) or comorbidities, such as DM (7.0% *vs*. 6.0%, *p* = 0.813) and HT (15.2% *vs*. 18.0%, *p* = 0.635). There were also no significant differences in the characteristics of the stones, such as their location, size, and density, between the two groups, except for Guy’s stone score and the degree of hydronephrosis. The multi-tract group had a higher Guy’s stone score (2.70 ± 0.789 *vs*. 2.46 ± 0.819, *p* = 0.028) and a higher proportion of patients with moderate to severe hydronephrosis (54.0% *vs*. 37.3%, *p* = 0.037) than the single-tract group. Demographic data and stone parameters of study participants are summarized in Table 1.

**Table 1:** Demographic data and stone parameters of patients.

	Single-tract (*n* = 158)	Multi-tract (*n* = 50)	p*
**Age (years)**, mean ± SD	48.6 ± 14.07	49.2 ± 13.50	0.798^1^
**Gender**, *n* (%)			0.982^2^
Male	123 (77.9)	39 (78.0)	
Female	35 (22.1)	11 (22.0)	
**BMI (kg/m**^**2**^**)**, mean ± SD	24.6 ± 2.73	24.6 ± 2.70	0.964^1^
**ASA score**, median (IQR)	1 (1–2)	1 (1–2)	0.435^3^
1, *n* (%)	101 (63.9)	29 (58.0)	0.628^2^
2, *n* (%)	50 (31.6)	18 (36.0)	
3, *n* (%)	7 (4.4)	3 (6.0)	
**Comorbidity**, *n* (%)			
DM	11 (7.0)	3 (6.0)	0.813^2^
HT	24 (15.2)	9 (18.0)	0.635^2^
**Laterality**, *n* (%)			0.263^2^
Right	71 (44.9)	27 (54.0)	
Left	87 (55.1)	23 (46.0)	
**History of ipsilateral renal ESWL**, *n* (%)	32 (20.3)	10 (20.0)	0.969^2^
**History of ipsilateral renal surgery**, *n* (%)			0.205^2^
None	112 (73.7)	40 (90.0)	
URS or RIRS	30 (19.0)	9 (18.0)	
PNL	5 (3.2)	1 (2.0)	
Open	14 (8.9)	0 (0.0)	
**Stone location**, *n* (%)			0.435^2^
Multiple calyces	13 (8.2)	7 (14.0)	
Pelvic and calix	116 (73.4)	33 (66.0)	
Staghorn	29 (18.4)	10 (20.0)	
**Guy’s stone score**, mean ± SD, (M)	2.46 ± 0.819 (M: 2.0)	2.70 ± 0.789 (M: 2.5)	**0.028** **^3^**
1, *n* (%)	4 (2.5)	0 (0)	**0.014** **^2^**
2, *n* (%)	106 (67.1)	25 (50.0)	
3, *n* (%)	19 (12.0)	15 (30.0)	
4, *n* (%)	29 (18.4)	10 (20.0)	
**Hydronephrosis**, median (IQR)	1 (1–2)	2 (1–2)	**0.034** **^3^**
None or mild, *n* (%)	99 (62.7)	23 (46.0)	**0.037** **^2^**
Moderate or severe, *n* (%)	59 (37.3)	27 (54.0)	
**Stone burden (mm**^**2**^**)**, mean ± SD, (M)	828.58 ± 627.19 (M: 609.5)	797.48 ± 496.49 (M: 645)	0.734^3^
**Stone density in Hounsfield units**, mean ± SD, (M)	1,190.92 ± 391.17 (M: 1,180)	1,263.64 ± 409.89 (M: 1,236)	0.261^3^

**Notes:**

ASA, American Society of Anesthesiologists; BMI, Body mass index; DM, Diabetes mellitus; ESWL, Extracorporeal shock wave lithotripsy; HT, Hypertension; PNL, Percutaneous nephrolithotomy; RIRS, Retrograde intrarenal surgery; URS, Ureteroscopy; IQR, Interquartile range; SD, Standard deviation; M, Median.

* The bold *p* values indicate *p* < 0.05, which is considered a statistically significant difference.

1 Independent samples T-test.

2 Chi-square test.

3 Mann-Whitney U test.

The average total fluoroscopy time was 1.7 ± 0.85 min in the single-tract group and 5.8 ± 2.21 min in the multi-tract group, and the average total operation time was 73.59 ± 44.08 min in the single-tract group and 89.3 ± 27.99 min in the multi-tract group. The average total fluoroscopy time and the average total operation time were both significantly longer in the multi-tract group than in the single-tract group (*p* < 0.001). There were no statistically significant differences in preoperative and postoperative hemoglobin levels between the two groups; however, the multi-tract group experienced a greater mean and percentage drop in hemoglobin (2.0 ± 0.99 g/dl and 14.0% *vs*. 1.7 ± 1.16 g/dl and 12.2%, *p* = 0.027 and *p* = 0.017, respectively). Despite this, there was no significant difference in transfusion rates between the two groups (7.6% *vs*. 12.0%, *p* = 0.334).

The time to nephrostomy catheter removal was comparable in both groups (*p* = 0.215). However, the length of hospital stay was significantly higher in the multi-tract group than in the single-tract group (4.2 ± 1.52 days *vs*. 3.7 ± 1.49 days, *p* = 0.018). There was no statistically significant difference in stone-free rates between the two groups (76.0% *vs*. 78.0%, *p* = 0.766). All intraoperative findings and postoperative outcomes are detailed in Table 2.

**Table 2:** Intraoperative findings and postoperative outcomes.

	Single-tract (*n* = 158)	Multi-tract (*n* = 50)	p*
**Total fluoroscopy time (min)**, mean ± SD, (M)	1.7 ± 0.85 (M: 1.4)	5.8 ± 2.21 (M: 6.0)	**<0.001** **^1^**
**Total operation time (min)**, mean ± SD, (M)	73.6 ± 44.08 (M: 59.5)	89.3 ± 27.99 (M: 80.0)	**<0.001** **^1^**
**Preoperative HGB levels (g/dl)**, mean ± SD, (M)	14.3 ± 1.57 (M: 14.5)	14.2 ± 1.71 (M: 14.6)	0.949^1^
**Postoperative HGB levels (g/dl)**, mean ± SD, (M)	12.6 ± 1.75 (M: 12.8)	12.2 ± 1.48 (M: 12.1)	0.113^1^
**HGB drop (g/dl)**, mean ± SD, (M)	1.7 ± 1.16 (M: 1.6)	2.0 ± 0.99 (M: 2.0)	**0.027** **^1^**
**HGB drop (%)**, mean ± SD, (M)	12.2 ± 7.78 (M: 11.0)	14.0 ± 6.25 (M: 14.2)	**0.017** **^1^**
**Blood transfusion**, *n* (%)	12 (7.6)	6 (12.0)	0.334^2^
**Stone free**, *n* (%)	120 (76.0)	39 (78.0)	0.766^2^
**Time to nephrostomy catheter removal (day)**, mean ± SD, (M)	2.7 ± 0.96 (M: 3.0)	2.9 ± 1.04 (M: 3.0)	0.215^1^
**Hospital stay length (day)**, mean ± SD, (M)	3.7 ± 1.49 (M: 4.0)	4.2 ± 1.52 (M: 4.0)	**0.018** **^1^**
**Clavien-Dindo classification of complications**, *n* (%)			0.896^2^
Grade 1	4 (2.5)	1 (2.0)	
Grade 2	16 (10.1)	7 (14.0)	
Grade 3	5 (3.2)	2 (4.0)	

**Notes:**

HGB, Hemoglobin; SD, Standard deviation; M, Median.

* The bold *p* values indicate *p* < 0.05, which is considered a statistically significant difference.

1 Mann-Whitney U test.

2 Chi-square test.

A total of 20 (12.6%) patients in the single-tract group and eight (16%) patients in the multi-tract group experienced minor complications, including postoperative fever, urinary tract infection (UTI), and blood transfusion. Five (3.2%) patients in the single tract group experienced major complications, including three ureterorenoscopy due to migrated stones, one double J stent insertion due to prolonged urinary leak after the removal of the nephrostomy catheter, and one selective angioembolization due to uncontrolled bleeding. Two (4%) patients in the multi-tract group experienced pneumothorax, a major complication, and required chest tube placement. After the symptoms of these two patients improved, the chest tubes were removed, and they were discharged. There was no statistically significant difference in complications between the two groups, and no Clavien Grade 4 or Grade 5 complications were observed in either group (*p* = 0.896).

## Discussion

Kidney stones are a prevalent urological issue and carry a high risk of recurrence (Jiao et al., 2020). In addition to their potential to impair kidney function, large kidney stones can lead to recurrent infections and life-threatening complications such as sepsis. Therefore, it is crucial to ensure the complete clearance of large and staghorn stones (Ganpule & Desai, 2008). PNL is a preferred treatment for large kidney stones, but achieving complete stone clearance may require multiple access points (Zhou et al., 2017). While many previous studies have shown the safety of multi-tract PNL compared to single-tract PNL in the treatment of large renal stones, the results remain arguable (Jiao et al., 2020). In this study, we did not establish a specific criterion for determining preoperative multi-track preference due to the study’s retrospective design. There was no statistically significant difference between the groups for stone burden. However, the higher Guy’s Stone scores in the multi-track group may be a measurable reason for our preference for multi-track. Additionally, this finding suggests that evaluating a patient’s Guy’s stone score before surgery may also help the surgeon predict the preferred method in clinical practice.

Achieving a stone-free status is the main objective of PNL. In their study, Singla et al. (2008) reported a success rate of 70% in achieving a stone-free status with multi-tract access after a single PNL session involving a median of three accesses in 164 renal units. In a prospective randomized study, Zhong et al. (2011) compared single-tract and multi-tract access PNL in 54 patients with staghorn stones. They demonstrated that multiple access PNL was better at stone clearance and resulted in a reduced need for additional procedures after surgery compared to single-tract PNL (Zhong et al., 2011). In another study, Akman et al. (2010) compared the early outcomes of single-tract PNL *vs*. multi-tract PNL in the treatment of staghorn stones. This retrospective study revealed a stone-free rate of 70.1% in the single-tract group and 81.1% in the multi-tract group (Akman et al., 2010). In their meta-analysis, Jiao et al. (2020) found no significant difference in the stone-free rate between single-tract and multi-tract PNL. They suggested that the variability in stone-free rates may be because there is no universally accepted definition of stone-free rate across studies and different studies used different imaging modalities for postoperative assessments (KUB radiography, ultrasound, or CT). They also suggested that the timing of patient follow-up may have impacted study results, as the final stone-free rate is often higher than the immediate postoperative stone-free rate due to the time required for stone fragments to be naturally expelled through urine. In the present study, all patients were re-evaluated using non-contrast abdominal tomography in the first month after surgery, and the results also showed comparable stone clearance rates between the single-tract and multi-tract groups.

There is a concern that creating multiple percutaneous accesses may increase the risk of bleeding and complications compared to procedures requiring a single access (Kukreja et al., 2004). However, some studies have shown that multiple tracts do not significantly increase the risk of bleeding or the need for blood transfusions (Ganpule, Mishra & Desai, 2009; Hegarty & Desai, 2006). Notably, these studies found that the need for transfusion is associated with low preoperative hemoglobin levels. Similarly, Auge, Dahm & Bach (2001) found no significant difference in blood loss, transfusion rates, complications, or operative duration between single-tract and multi-tract PNL. The AUA nephrolithiasis guidelines panel on staghorn calculi reported 7–27% complication rates and a transfusion rate of up to 18% (Preminger et al., 2005). A recent meta-analysis reported that the blood transfusion rate was higher in patients with multi-tract PNL than in patients with single-tract PNL, but the rates of other complications did not significantly differ between the groups (Jiao et al., 2020). These differing results may be due to the experience level of the surgeon and the surgical technique used during multi-tract PNL (Hegarty & Desai, 2006). Morbidity can be reduced with PNL if the surgeon always punctures in full expiration, stays in the lateral half of the rib, and uses a working sheath during nephroscopy and a well-draining nephrostomy tube after the procedure (Sukumar et al., 2008). In the present study, despite a larger decrease in hemoglobin levels seen in the multi-tract group than the single-tract group, there was no statistically significant difference in transfusion rates between the two groups. Although a drop in hemoglobin levels was much more common in the multi-tract group, selective angioembolization was performed on one patient in the single-tract group due to uncontrollable bleeding. Longer fluoroscopy and operation times were seen in the multi-tract PNL group than in the single-tract PNL group in the current study, but using ultrasound guidance in multi-tract PNL may help eliminate these differences. Ultrasound guidance has several advantages, such as the absence of ionizing radiation, a shorter procedure time, fewer punctures, and does not require contrast agents (Arabzadeh Bahri et al., 2023).

Secondary accesses in PNL are mainly used to access stones that cannot be reached in the upper calyx. Since these approaches often require an intercostal approach, caution should be exercised regarding pulmonary complications (Shah et al., 2006; Sukumar et al., 2008). Pulmonary damage due to lung transgression can occur in any location, ranging from 14% on the left side to as high as 29% on the right side, even during controlled expirations (Hopper & Yakes, 1990). In the present study, two patients in the multi-tract group who developed pneumothorax were treated with chest tube insertion. To reduce these complications, patients who will require a second access should be identified through appropriate preoperative evaluation, and the second access should be created before securing the guide wires (El-Nahas et al., 2012).

This study has several limitations that should be acknowledged. First, the retrospective design may introduce inherent biases that could affect the validity of the findings. Second, the single-center nature of the study, with operations performed by a single surgeon, limits the generalizability of the results to broader populations. Third, the unequal sample sizes between the groups may have influenced the statistical power and comparability of the outcomes. Lastly, the absence of data on the composition of the stones limited the ability to assess the impact of stone characteristics on surgical outcomes.

## Conclusions

The findings of this study suggest that multiple-access tracts result in high stone-free rates and a slight increase in the incidence of acceptable complications. To avoid the higher surgical risks and expenses associated with multiple procedures, it would be advantageous to achieve stone-free results in a single session. Therefore, urologists involved in percutaneous surgery should consider multiple accesses for hard-to-reach stones and should be capable of performing secondary accesses when necessary.

## Supplemental Information

10.7717/peerj.18450/supp-1 Supplemental Information 1 Parameters of study groups.

## Additional Information and Declarations

### Data Availability

The following information was supplied regarding data availability: Raw data, including the demographic variables, stone parameters, fluoroscopy and operation times and postoperative parameters of the study groups, are available as a Supplemental File.
